# A retrospective comparative study of ChatGPT-5.4 and DeepSeek-VL2 for C-TIRADS-based risk stratification of thyroid nodules on ultrasound images

**DOI:** 10.3389/fdgth.2026.1877420

**Published:** 2026-07-23

**Authors:** Danhong Cai, Shaopan Cai, Jiaxuan Ye, Zhirong Xu, Weipeng Li

**Affiliations:** 1 Department of Ultrasound, Jinjiang Municipal Hospital, Jinjiang, Fujian Province, China; 2 Department of Ultrasound, Jinjiang Municipal Hospital Jinnan Branch, Jinjiang, Fujian Province, China; 3 School of Public Health, Fujian Medical University, Fuzhou, Fujian Province, China; 4 Department of Ultrasound, The Second Affiliated Hospital of Fujian Medical University, Quanzhou, Fujian Province, China

**Keywords:** ChatGPT-5.4, C-TIRADS, DeepSeek-VL2, multimodal large language model, thyroid nodules, ultrasound

## Abstract

**Background/objectives:**

Multimodal large language models are increasingly being explored for medical image interpretation, but their performance in thyroid nodule ultrasound assessment remains uncertain. This study compared ChatGPT-5.4 and DeepSeek-VL2 for C-TIRADS-based risk stratification of thyroid nodules using static ultrasound images.

**Methods:**

This single-center retrospective diagnostic accuracy study included 206 pathologically confirmed thyroid nodules, including 117 benign and 89 malignant nodules. For each nodule, paired transverse and longitudinal ultrasound images were analyzed by both models using the same standardized English prompt. The models generated structured outputs for C-TIRADS-related ultrasound features and estimated C-TIRADS category. Pathology served as the reference standard for malignancy, and expert-assigned C-TIRADS served as the imaging reference standard. Model-assigned C-TIRADS categories were used as ordinal risk scores. The primary analysis used C-TIRADS ≥4B as the binary positive threshold for predicting pathology-confirmed malignancy, and a sensitivity analysis used C-TIRADS ≥4A

**Results:**

At the ≥4B threshold, ChatGPT-5.4 achieved higher accuracy, sensitivity, and specificity than DeepSeek-VL2: 70.9%, 61.8%, and 77.8% vs. 57.8%, 50.6%, and 63.2%, respectively. Its non-diagnostic rate was also lower (6.3% vs. 15.5%). In the valid-output ROC analysis, AUCs were 0.903 for expert assessment, 0.790 for ChatGPT-5.4, and 0.757 for DeepSeek-VL2; model-based ROC analyses excluded indeterminate outputs and should be interpreted as per-protocol estimates. At the C-TIRADS category level, ChatGPT-5.4 showed higher exact agreement with expert assessment than DeepSeek-VL2 (45.6% vs. 36.4%) and higher within-one-category agreement (87.6% vs. 82.2%). Feature-level analysis showed that both models performed best on echogenicity but poorly on margin and echogenic foci. At the ≥4A threshold, sensitivity increased but specificity decreased substantially for both models.

**Conclusions:**

ChatGPT-5.4 showed a more favorable performance profile than DeepSeek-VL2, but differences in sensitivity and valid-output AUC were not statistically significant. Both models remain investigational adjunctive tools and should not be used for unsupervised clinical decision-making or standalone triage.

## 1 Introduction

Thyroid nodules are among the most common endocrine imaging findings encountered in clinical practice (1, 2). Ultrasound is the preferred first-line imaging modality for initial evaluation, risk stratification, and follow-up, while fine-needle aspiration remains central to deciding whether surgical management is warranted in selected nodules. Suspicious sonographic features, including solid composition, hypoechogenicity, irregular margins, and punctate echogenic foci, play a key role in estimating malignancy risk (1, 2).

To standardize ultrasound-based risk assessment, several thyroid imaging reporting and data systems have been developed. In China, the 2020 Chinese guidelines for ultrasound malignancy risk stratification of thyroid nodules introduced the C-TIRADS system, which provides a structured framework for classifying nodules according to malignant sonographic features and guiding further management (3). Because C-TIRADS is an ordinal risk stratification system rather than a simple binary classifier, different thresholds such as category 4A or 4B may serve distinct clinical purposes depending on whether sensitivity or specificity is prioritized.

Meanwhile, multimodal large language models have rapidly emerged as a new class of general-purpose systems capable of integrating text and image inputs (4, 5). Compared with conventional task-specific deep learning models, these models offer greater flexibility and stronger interaction capabilities, but their performance in highly specialized medical imaging tasks remains incompletely understood. This issue is especially relevant in thyroid ultrasound, where interpretation depends not only on image-level classification but also on subtle, fine-grained visual features and structured risk stratification logic. Recent studies have begun to explore multimodal GPT systems in thyroid nodule assessment, including ChatGPT-4 Vision for ultrasound-based C-TIRADS analysis and ThyGPT, a domain-specific multimodal GPT model trained for thyroid nodule diagnosis and management (4, 6–8). These studies suggest that GPT-based systems are feasible for thyroid ultrasound tasks, but they also indicate that general multimodal models remain below expert performance and may struggle with detailed sonographic reasoning. ChatGPT-5.4 and DeepSeek-VL2 were selected because they are accessible general-purpose multimodal systems that represent different model ecosystems and are likely to be encountered by clinicians, researchers, and patients in real-world settings. In contrast, thyroid-specific systems such as ThyGPT represent a domain-adapted development pathway and were therefore considered contextual comparators rather than the main targets of this head-to-head evaluation.

However, direct real-world comparisons between different general-purpose multimodal LLMs remain limited, especially under identical imaging data, prompt design, and structured C-TIRADS output requirements. This gap is clinically important because thyroid ultrasound interpretation requires not only binary benign-vs.-malignant judgment but also recognition of fine-grained sonographic features and stable ordinal risk stratification. Therefore, we conducted a retrospective comparative study using pathologically confirmed thyroid nodules from Jinjiang Municipal Hospital. By using paired transverse and longitudinal static ultrasound images, the same standardized prompt, and the same C-TIRADS-oriented structured output scheme, we compared ChatGPT-5.4 and DeepSeek-VL2 in terms of C-TIRADS-based risk stratification, threshold-based prediction of pathology-confirmed malignancy, agreement with expert C-TIRADS classification, feature-level recognition, and non-diagnostic output.

## 2 Materials and methods

### 2.1 Study design and study population

**Figure 1:**
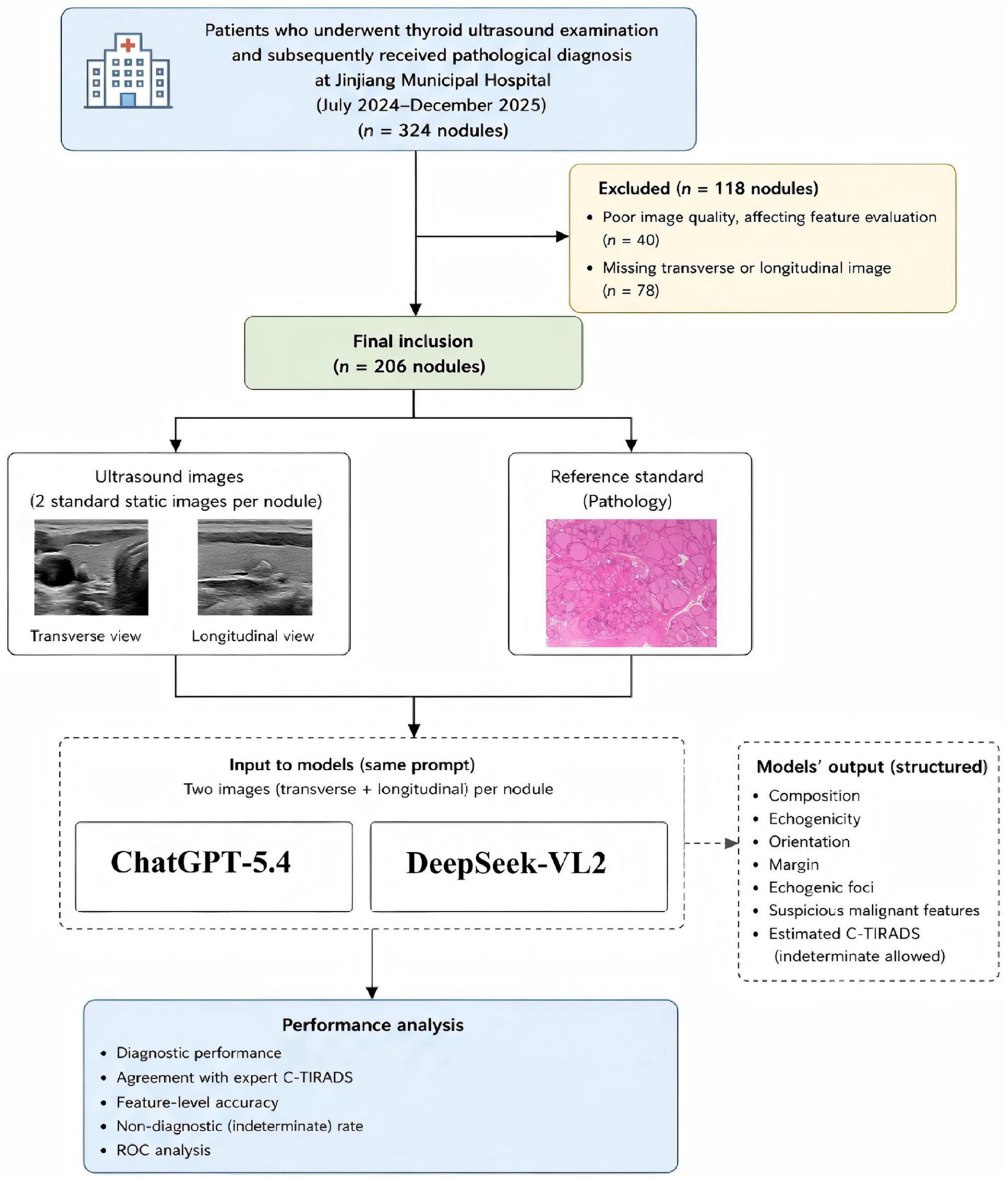
Flowchart of patient and nodule selection. A total of 324 thyroid nodules were initially screened. After exclusion of 118 nodules that did not meet the eligibility criteria, including 40 with insufficient image quality and 78 lacking either a transverse or longitudinal ultrasound image, 206 nodules were finally included in the analysis.

This was a single-center retrospective diagnostic accuracy study. We consecutively included patients who underwent thyroid ultrasound at Jinjiang Municipal Hospital between July 2024 and December 2025 and had subsequent pathological confirmation. A total of 324 thyroid nodules were initially screened. Of these, 118 were excluded, including 40 due to insufficient image quality for reliable sonographic interpretation and 78 due to the absence of either a transverse or longitudinal ultrasound image. Ultimately, 206 thyroid nodules were included in the final analysis (Figure 1). This study was approved by the Ethics Committee of Jinjiang Municipal Hospital (approval number: JJSYYLL-2026-066). The requirement for informed consent was waived due to the retrospective nature of the study and the use of anonymized data.

Patients were included if they had available preoperative ultrasound images of the same thyroid nodule in both transverse and longitudinal planes, a definitive pathological diagnosis, and clear correspondence between the images and the target lesion. Cases were excluded if image quality was insufficient to allow reliable assessment of key sonographic features, or if either the transverse or longitudinal image was missing. Insufficient image quality was defined as severe image blurring, incomplete visualization of the target nodule, substantial acoustic artifacts, unclear lesion boundaries, or inability to reliably assess key C-TIRADS-related sonographic features. Image quality was assessed by the expert sonographer panel before model evaluation.

### 2.2 Ultrasound images and reference standards

Each included nodule was evaluated using two static ultrasound images of the same lesion, consisting of one transverse view and one longitudinal view. Pathology served as the reference standard for benign-vs.-malignant classification.

The imaging reference standard was expert-assigned C-TIRADS classification. The expert imaging reference standard was established by three board-certified sonographers, each with more than 10 years of experience in thyroid ultrasound. The sonographers first reviewed the ultrasound images independently and then discussed discrepant feature assessments and C-TIRADS categories until consensus was reached. All expert readers were blinded to the pathological diagnosis and to the outputs of ChatGPT-5.4 and DeepSeek-VL2. During consensus review, the readers assessed the five core C-TIRADS-related ultrasound features, including composition, echogenicity, orientation, margin, and echogenic foci, and then assigned the final expert C-TIRADS category according to the 2020 Chinese C-TIRADS guideline.

### 2.3 LLM prompting strategy

For each nodule, the transverse and longitudinal images were provided to each model as a paired image set. The standardized English prompt explicitly instructed the model to analyze both images jointly as the same lesion rather than making independent judgments from a single view. The models were required to provide structured outputs for composition, echogenicity, orientation, margin, echogenic foci, suspicious malignant features, and an estimated C-TIRADS category. This structured, image-pair-based prompting approach was consistent with emerging multimodal thyroid nodule studies that use task-specific prompts and C-TIRADS-oriented outputs.

ChatGPT-5.4 was accessed through the web-based interface between April 1 and April 30, 2026. The open-source DeepSeek-VL2 model, released on December 13, 2024, was accessed during the same evaluation period via third-party cloud-hosted application programming interface (API) services for image inference. Each nodule was evaluated in a fresh session to minimize contextual carryover from previous cases. Only the paired transverse and longitudinal ultrasound images were provided to the models; no clinical information, pathological results, or expert C-TIRADS labels were included in the input. For ChatGPT-5.4, sampling parameters such as temperature and top-*p* were not user-controllable through the web interface. For DeepSeek-VL2, the default API inference settings were used without manual adjustment of sampling parameters. Each case was evaluated once by each model, and repeat testing was not performed.

If a model could not confidently determine a given feature based on the available images, the output for that feature was recorded as “indeterminate.” If any required feature for C-TIRADS estimation was indeterminate, the final estimated C-TIRADS category was also recorded as indeterminate.

The full standardized English prompt is provided in Supplementary Material 1. The prompt required the models to analyze transverse and longitudinal images jointly, output structured C-TIRADS-related sonographic features, and avoid direct benign/malignant diagnosis or management recommendations.

### 2.4 Outcome definitions

The primary outcome was pathology-based diagnostic performance for predicting malignancy. Because C-TIRADS is an ordinal risk stratification system and category 4A and 4B represent clinically distinct risk thresholds, model-assigned C-TIRADS categories were used as ordinal risk scores. We prespecified two binary positive thresholds for predicting pathology-confirmed malignancy: estimated C-TIRADS ≥4B for the primary analysis and estimated C-TIRADS ≥4A for the sensitivity analysis. These thresholds were not regarded as definitions of malignancy itself.

Secondary outcomes included agreement between model-assigned and expert-assigned C-TIRADS categories, feature-level performance for the five core sonographic features, and the frequency of indeterminate outputs, which was defined as the non-diagnostic rate. At the feature level, indeterminate outputs were treated as failed classifications. At the C-TIRADS level, indeterminate outputs were treated as failed categorizations and were counted as incorrect in the primary binary diagnostic analysis, while also being reported separately as non-diagnostic outputs.

### 2.5 Statistical analyses

Diagnostic performance metrics included accuracy, sensitivity, specificity, positive predictive value, negative predictive value, and F1 score. ROC curves were generated using ordinal C-TIRADS categories as risk scores, with expert assessment shown as a reference comparator. Agreement analyses included exact agreement, valid-case exact agreement, within-one-category agreement, and weighted kappa. Feature-level accuracy and indeterminate rates were summarized using heatmaps, and the relationship between expert-assigned and model-assigned C-TIRADS categories was visualized using agreement heatmaps and category-distribution plots. The study design and reporting structure were aligned with the principles of STARD 2015 and are also conceptually consistent with the recently published STARD-AI guidance for AI-centered diagnostic accuracy studies. All statistical analyses were performed using Python version 3.8.0. Where applicable, 95% confidence intervals were calculated. For ROC analysis, cases with indeterminate C-TIRADS outputs could not be assigned ordinal risk scores and were therefore excluded from the ROC curves. Accordingly, the model-based ROC analysis was defined as a valid-output, per-protocol analysis rather than a full-cohort analysis. This resulted in 193 valid cases for ChatGPT-5.4 and 174 valid cases for DeepSeek-VL2. Because both models were evaluated on the same nodules, paired comparisons were performed. McNemar's exact test was used to compare paired proportions, including accuracy, sensitivity, specificity, and non-diagnostic rates. The paired statistical comparisons are summarized in Supplementary Table S2. For ROC analysis, DeLong's test was used to compare correlated AUCs on the common valid-output subset in which both models produced valid ordinal C-TIRADS scores. All statistical tests were two-sided, and *p* < 0.05 was considered statistically significant. Paired statistical comparisons were not performed for PPV, NPV, or F1 score because these summary measures are not simple paired proportions.

To explore whether indeterminate outputs were associated with observable case characteristics, we compared valid-output and indeterminate-output nodules for each model using available clinical and imaging variables, including age, sex, maximum nodule diameter, pathological outcome, and expert-assigned C-TIRADS category. Age and maximum nodule diameter were compared using the Mann–Whitney U test, sex and pathological outcome were compared using Fisher's exact test, and expert C-TIRADS distributions were compared using the chi-square test and interpreted descriptively because of small cell counts. Quantitative image-quality scores were not available because all included nodules had already passed the predefined image-quality screening before model evaluation.

## 3 Results

### 3.1 Study population

The final cohort included 206 pathologically confirmed thyroid nodules, consisting of 117 benign nodules and 89 malignant nodules. The mean patient age was 47.60 ± 13.07 years, and the mean maximum nodule diameter was 14.11 ± 4.98 mm. Expert-assigned C-TIRADS categories were mainly distributed across categories 3, 4A, and 4B, indicating that the cohort included low-, intermediate-, and relatively high-risk nodules. The exclusion process is summarized in Figure 1.

### 3.2 Prediction of pathology-confirmed malignancy using C-TIRADS thresholds

In the primary analysis using model-assigned C-TIRADS ≥4B as the binary positive threshold for predicting pathology-confirmed malignancy, ChatGPT-5.4 showed higher point estimates than DeepSeek-VL2 across the main binary diagnostic metrics. ChatGPT-5.4 achieved an accuracy of 70.9%, sensitivity of 61.8%, and specificity of 77.8%, whereas DeepSeek-VL2 achieved 57.8%, 50.6%, and 63.2%, respectively. The non-diagnostic rate was lower for ChatGPT-5.4 than for DeepSeek-VL2 (6.3% vs. 15.5%). In paired comparisons, ChatGPT-5.4 showed significantly higher accuracy than DeepSeek-VL2 at the primary ≥4B threshold (McNemar's exact test, *p* = 0.005), mainly driven by higher specificity (*p* = 0.009), whereas the difference in sensitivity was not statistically significant (*p* = 0.193). The full diagnostic performance metrics are summarized in Table 1.

**Table 1:** Diagnostic performance of model-assigned C-TIRADS thresholds for predicting pathology-confirmed malignancy.

Metric	ChatGPT-5.4	DeepSeek-VL2
**Primary analysis (threshold ≥4B)**		
Accuracy	70.9% (64.3–76.7)	57.8% (50.9–64.3)
Sensitivity	61.8% (51.4–71.2)	50.6% (40.4–60.7)
Specificity	77.8% (69.4–84.4)	63.2% (54.2–71.4)
PPV	67.9% (57.1–77.1)	51.1% (40.9–61.3)
NPV	72.8% (64.4–79.8)	62.7% (53.7–70.9)
F1 score	64.7% (55.6–72.7)	50.8% (41.1–59.8)
Non-diagnostic rate	6.3% (3.7–10.5)	15.5% (11.2–21.1)
**Sensitivity analysis (threshold ≥4A)**		
Accuracy	58.3% (51.4–64.8)	52.4% (45.6–59.1)
Sensitivity	84.3% (75.3–90.4)	73.0% (63.0–81.2)
Specificity	38.5% (30.1–47.5)	36.8% (28.6–45.8)
PPV	51.0% (43.0–59.0)	46.8% (38.7–55.0)
NPV	76.3% (64.0–85.3)	64.2% (52.2–74.6)
F1 score	63.6% (55.8–70.4)	57.0% (48.8–64.4)
Non-diagnostic rate	6.3% (3.7–10.5)	15.5% (11.2–21.1)

This table summarizes the diagnostic performance metrics under the primary binary positive threshold of C-TIRADS ≥4B and the sensitivity-analysis threshold of C-TIRADS ≥4A for predicting pathology-confirmed malignancy. These thresholds were used as positive prediction cutoffs and were not considered definitions of malignancy itself.

**Figure 2:**
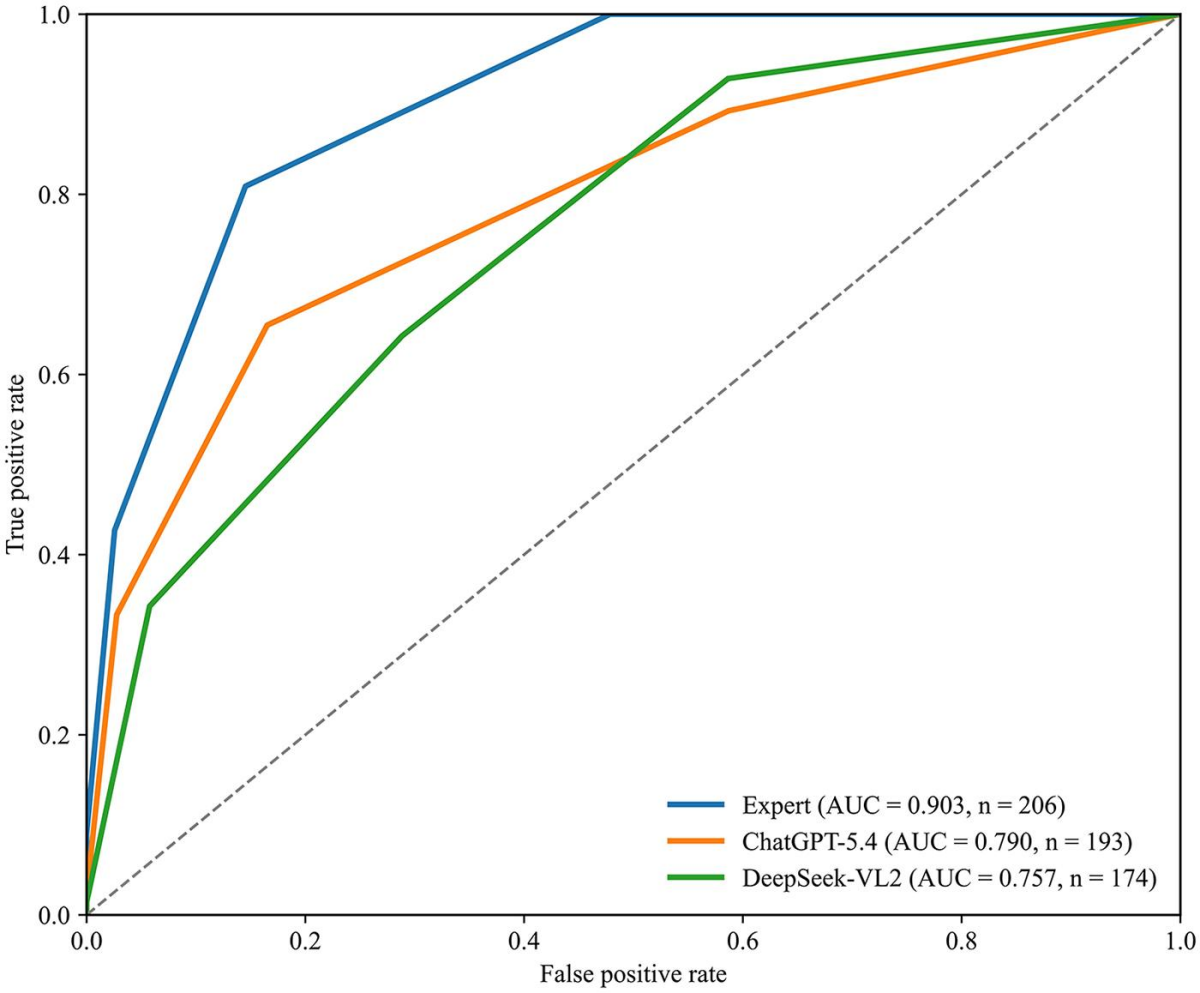
ROC curves for predicting pathology-confirmed malignancy using ordinal C-TIRADS scores. ROC curves were generated using ordinal C-TIRADS categories as risk scores. The model-based ROC curves were based on valid-output cases only because indeterminate C-TIRADS outputs could not be assigned ordinal risk scores. The AUC was 0.903 for expert assessment (*n* = 206), 0.790 for ChatGPT-5.4 (*n* = 193), and 0.757 for DeepSeek-VL2 (*n* = 174). On the common valid-output subset for both models (*n* = 164), the AUC was 0.785 for ChatGPT-5.4 and 0.765 for DeepSeek-VL2, with no statistically significant difference by DeLong's test.

In the valid-output ROC analysis, the AUC was 0.903 for expert assessment (*n* = 206), 0.790 for ChatGPT-5.4 (*n* = 193), and 0.757 for DeepSeek-VL2 (*n* = 174). Because indeterminate outputs were excluded from the model-based ROC curves, these AUCs should be interpreted as per-protocol estimates among cases with valid ordinal C-TIRADS outputs rather than full-cohort diagnostic performance (Figure 2). For the paired AUC comparison, DeLong's test was performed on the common valid-output subset, defined as cases in which both models produced valid ordinal C-TIRADS outputs (*n* = 164). In this subset, the AUC was 0.785 for ChatGPT-5.4 and 0.765 for DeepSeek-VL2, with no statistically significant difference (*p* = 0.680).

When the binary positive threshold was broadened from C-TIRADS ≥4B to C-TIRADS ≥4A in the sensitivity analysis, both models demonstrated higher sensitivity and lower specificity. For ChatGPT-5.4, sensitivity increased from 61.8% to 84.3%, whereas specificity decreased from 77.8% to 38.5%. For DeepSeek-VL2, sensitivity increased from 50.6% to 73.0%, whereas specificity decreased from 63.2% to 36.8%. These findings indicate a threshold-dependent shift toward higher detection of suspicious nodules at the cost of increased false positives. At the ≥4A threshold, the difference in accuracy between ChatGPT-5.4 and DeepSeek-VL2 was not statistically significant (*p* = 0.207). The sensitivity difference showed a numerical trend favoring ChatGPT-5.4 but did not reach statistical significance (*p* = 0.087), and specificity was similar between the two models (*p* = 0.885).

### 3.3 Agreement with expert C-TIRADS categorization

ChatGPT-5.4 showed better overall agreement with expert-assigned C-TIRADS categories than DeepSeek-VL2. The exact agreement rate was 45.6% for ChatGPT-5.4 and 36.4% for DeepSeek-VL2. After excluding non-diagnostic cases, the valid-case exact agreement rates were 48.7% and 43.1%, respectively. The within-one-category agreement rates were 87.6% for ChatGPT-5.4 and 82.2% for DeepSeek-VL2, with weighted kappa values of 0.584 and 0.502, respectively. The agreement metrics are summarized in Table 2.

**Table 2:** Agreement analysis between model-assigned and expert-assigned C-TIRADS categories.

Metric	ChatGPT-5.4	DeepSeek-VL2
Exact agreement	45.6% (39.0–52.5)	36.4% (30.1–43.2)
Valid-case exact agreement	48.7% (41.7–55.7)	43.1% (36.0–50.5)
Within-one-category agreement	87.6% (82.2–91.5)	82.2% (75.8–87.2)
Non-diagnostic rate	6.3% (3.7–10.5)	15.5% (11.2–21.1)
Weighted kappa (valid cases only)	0.584	0.502

This table presents agreement metrics between model-assigned and expert-assigned C-TIRADS categories, including exact agreement, valid-case exact agreement, within-one-category agreement, weighted kappa, and non-diagnostic rate.

**Figure 3:**
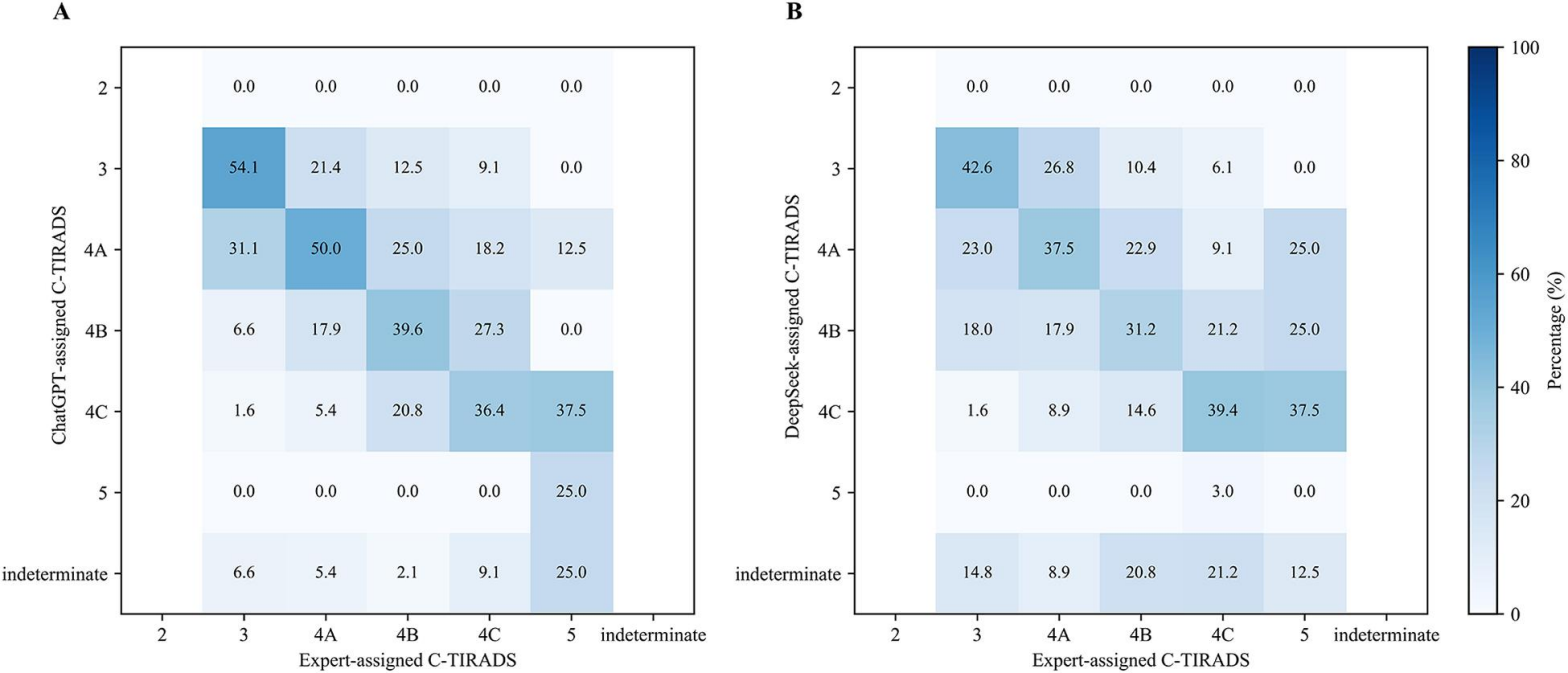
Heatmaps of agreement between model-assigned and expert-assigned C-TIRADS categories. Panel **(A)** shows the agreement heatmap between ChatGPT-5.4-assigned and expert-assigned C-TIRADS categories, and Panel **(B)** shows the corresponding heatmap for DeepSeek-VL2. Color intensity represents the percentage of each category pair. Most misclassifications occurred within adjacent categories. ChatGPT-5.4 showed relatively greater clustering around categories 3, 4A, and 4B, whereas DeepSeek-VL2 showed more category shifts and a higher proportion of indeterminate outputs.

**Figure 4:**
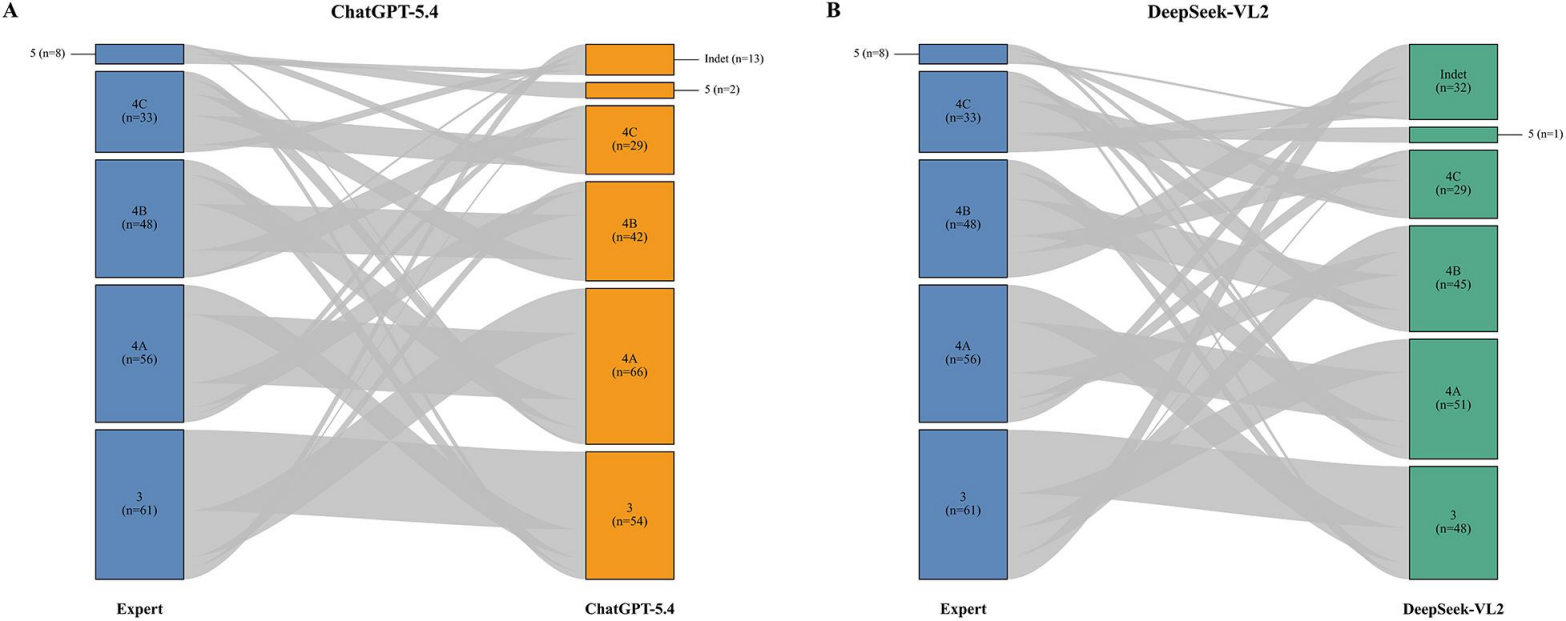
Overall distribution of C-TIRADS categories and indeterminate outputs for expert assessment and the two models. Panel **(A)** shows the overall distribution of expert-assigned and ChatGPT-5.4-assigned C-TIRADS categories, whereas Panel **(B)** shows the corresponding distribution for DeepSeek-VL2. ChatGPT-5.4 produced 13 indeterminate outputs, compared with 32 for DeepSeek-VL2, indicating that the latter was more likely to generate non-diagnostic outputs during structured risk stratification.

The agreement heatmaps further showed that most disagreements occurred between adjacent C-TIRADS categories rather than as large cross-category errors. ChatGPT-5.4 demonstrated tighter clustering around expert categories 3, 4A, and 4B, whereas DeepSeek-VL2 exhibited more frequent shifts to neighboring categories and more indeterminate outputs (Figure 3). At the overall category-distribution level, ChatGPT-5.4 produced 13 indeterminate outputs, whereas DeepSeek-VL2 produced 32, again indicating lower structural stability for DeepSeek-VL2 (Figure 4).

### 3.4 Feature-level performance

**Figure 5:**
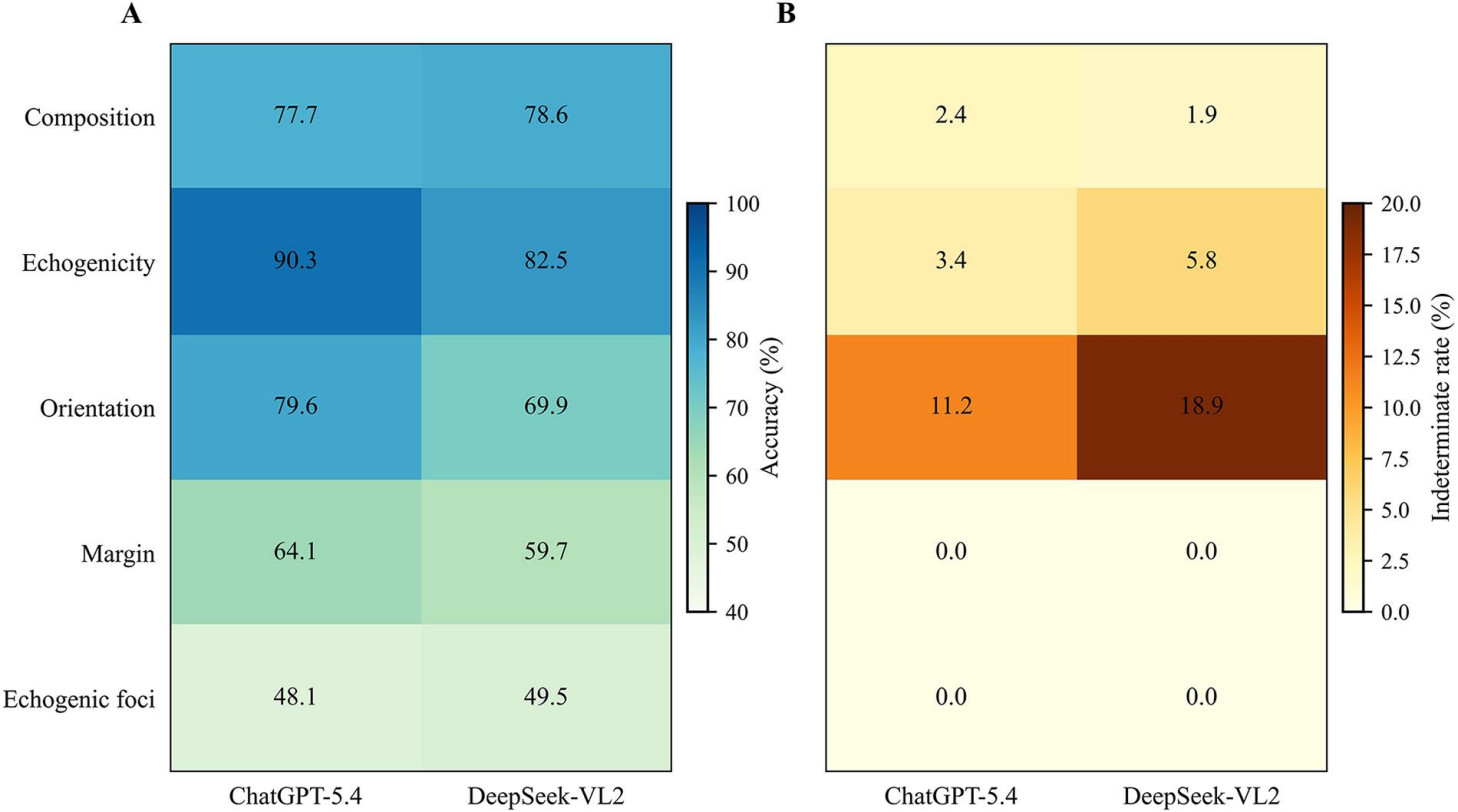
Heatmaps of feature-level accuracy and indeterminate rates for thyroid nodule ultrasound assessment. Panel **(A)** shows the feature-level accuracy of ChatGPT-5.4 and DeepSeek-VL2 for the five core ultrasound features, including composition, echogenicity, orientation, margin, and echogenic foci. Panel **(B)** shows the corresponding indeterminate rates. Both models performed best on echogenicity, whereas performance on margin and echogenic foci was relatively limited. Orientation had the highest indeterminate rate.

The two models showed substantial variation across the five core ultrasound features. ChatGPT-5.4 performed best on echogenicity, with an accuracy of 90.3%, followed by orientation at 79.6% and composition at 77.7%. Its accuracy was lower for margin (64.1%) and echogenic foci (48.1%). DeepSeek-VL2 also performed best on echogenicity, with an accuracy of 82.5%, followed by composition (78.6%) and orientation (69.9%), whereas its performance on margin and echogenic foci was 59.7% and 49.5%, respectively. Overall, both models performed relatively well on echogenicity but poorly on margin and echogenic foci (Figure 5A, Table 3).

**Table 3:** Feature-level performance of the two models for thyroid nodule ultrasound assessment.

Feature	ChatGPT-5.4 Accuracy	ChatGPT-5.4 Indeterminate rate	DeepSeek-VL2 Accuracy	DeepSeek-VL2 Indeterminate rate
Composition	77.7% (71.5–82.8)	2.4% (1.0–5.6)	78.6% (72.5–83.7)	1.9% (0.8–4.9)
Echogenicity	90.3% (85.5–93.6)	3.4% (1.7–6.8)	82.5% (76.8–87.1)	5.8% (3.4–9.9)
Orientation	79.6% (73.6–84.5)	11.2% (7.6–16.2)	69.9% (63.3–75.8)	18.9% (14.2–24.8)
Margin	64.1% (57.3–70.3)	0.0% (0.0–1.8)	59.7% (52.9–66.2)	0.0% (0.0–1.8)
Echogenic foci	48.1% (41.3–54.9)	0.0% (0.0–1.8)	49.5% (42.8–56.3)	0.0% (0.0–1.8)

This table summarizes the accuracy and indeterminate rate for the five core ultrasound features, including composition, echogenicity, orientation, margin, and echogenic foci.

Indeterminate outputs occurred mainly in orientation assessment. The orientation indeterminate rate was 11.2% for ChatGPT-5.4 and 18.9% for DeepSeek-VL2. Indeterminate outputs were rare for composition and echogenicity, and were not observed for margin or echogenic foci (Figure 5B).

To further examine whether correct final C-TIRADS categorization was supported by correct feature-level recognition, we cross-tabulated feature-level correctness against final C-TIRADS correctness (Table 4). For ChatGPT-5.4, only 44 nodules (21.4%) showed both exact final C-TIRADS agreement and correct recognition of all five ultrasound features, whereas 50 nodules (24.3%) had correct final C-TIRADS categorization despite at least one feature-level error. For DeepSeek-VL2, the corresponding numbers were 30 nodules (14.6%) and 45 nodules (21.8%). In addition, 37 ChatGPT-5.4 cases (18.0%) and 41 DeepSeek-VL2 cases (19.9%) showed incorrect final C-TIRADS categorization despite correct recognition of at least four features. These findings indicate that final C-TIRADS agreement alone may overestimate the models' true sonographic interpretation ability, because some correct final categories may reflect compensatory feature-level errors rather than fully accurate image understanding.

**Table 4:** Cross-tabulation of feature-level correctness and final C-TIRADS correctness.

Category	ChatGPT-5.4, *n* (%)	DeepSeek-VL2, *n* (%)
Final C-TIRADS correct and all five features correct	44 (21.4)	30 (14.6)
Final C-TIRADS correct despite ≥1 feature-level error	50 (24.3)	45 (21.8)
Final C-TIRADS incorrect despite ≥4 features correct	37 (18.0)	41 (19.9)
Final C-TIRADS incorrect with ≤3 features correct	62 (30.1)	58 (28.2)
Final C-TIRADS indeterminate	13 (6.3)	32 (15.5)

Data are presented as *n* (% of 206 nodules). Feature-level correctness was defined as exact agreement with expert assessment across the five core ultrasound features: composition, echogenicity, orientation, margin, and echogenic foci. Final C-TIRADS correctness was defined as exact agreement between model-assigned and expert-assigned C-TIRADS category. This table summarizes whether final C-TIRADS agreement was supported by fully correct feature-level recognition or may have reflected compensatory feature-level errors.

### 3.5 Non-diagnostic outputs

Among the 206 nodules, ChatGPT-5.4 generated 13 indeterminate C-TIRADS outputs, whereas DeepSeek-VL2 generated 32. Consistent with its more favorable performance profile and higher agreement estimates, ChatGPT-5.4 also showed greater stability in completing structured risk stratification. The non-diagnostic rate was significantly lower for ChatGPT-5.4 than for DeepSeek-VL2 in paired comparison (6.3% vs. 15.5%; McNemar's exact test, *p* = 0.003).

To assess whether indeterminate outputs were likely to be random, we compared valid-output and indeterminate-output nodules for each model using available case-level variables (Supplementary Table S3). For ChatGPT-5.4, no significant differences were observed between valid-output and indeterminate-output nodules in age, sex, maximum nodule diameter, pathological outcome, or expert-assigned C-TIRADS distribution. For DeepSeek-VL2, indeterminate-output nodules showed a numerically higher proportion of malignant pathology than valid-output nodules (19/32, 59.4% vs. 70/174, 40.2%; *p* = 0.053), although this difference did not reach statistical significance. No quantitative image-quality score was available because all included nodules had passed the predefined image-quality screening before model evaluation.

## 4 Discussion

In this retrospective comparative study of 206 pathologically confirmed thyroid nodules, ChatGPT-5.4 showed numerically better performance than DeepSeek-VL2 in structured C-TIRADS-based risk stratification and prediction of pathology-confirmed malignancy using predefined C-TIRADS thresholds. This pattern was observed across several endpoints, including overall diagnostic performance, agreement with expert-assigned C-TIRADS categories, and the ability to produce usable structured outputs. In the primary analysis using model-assigned C-TIRADS ≥4B as the binary positive threshold for predicting pathology-confirmed malignancy, ChatGPT-5.4 achieved higher accuracy, sensitivity, and specificity than DeepSeek-VL2, while also yielding fewer indeterminate outputs. However, paired statistical testing showed that the between-model difference was statistically significant for accuracy, specificity, and non-diagnostic rate, but not for sensitivity or valid-output AUC. These findings suggest that, under the present experimental design, ChatGPT-5.4 may provide more stable structured outputs than DeepSeek-VL2, although the between-model advantage was not statistically significant for all endpoints.

Beyond the overall performance pattern, our findings reveal a clear feature-dependent pattern in the performance of general multimodal LLMs for ultrasound interpretation. Both models performed relatively well on echogenicity and composition, which are comparatively global and visually salient image characteristics. By contrast, performance declined substantially for margin, echogenic foci, and orientation, which require finer appreciation of boundary irregularity, small punctate echoes, and spatial relationships across imaging planes. This pattern is aligned with recent thyroid nodule studies indicating that multimodal GPT-based systems can generate structured ultrasound assessments but remain limited in detailed feature recognition and risk stratification consistency (5, 7). In our study, this limitation was particularly evident for orientation, which had the highest indeterminate rate for both models. The result is clinically plausible, since orientation depends not only on lesion morphology but also on standardization of imaging planes and careful integration of information across views (1, 3). The cross-tabulation of feature-level correctness and final C-TIRADS correctness further supports this interpretation. A substantial proportion of nodules had correct final C-TIRADS categorization despite at least one feature-level error, indicating that final category agreement alone may overestimate the models' true sonographic interpretation ability. Therefore, feature-level verification remains necessary when evaluating multimodal LLMs for thyroid ultrasound assessment, particularly before considering any clinical use.

In addition to feature-level variability, model performance was also sensitive to the binary positive C-TIRADS threshold used to predict pathology-confirmed malignancy. When the positive threshold was broadened from C-TIRADS ≥4B to ≥4A, sensitivity increased for both models, but specificity dropped markedly. A lower threshold may be more appropriate if the model is used as an early screening aid prioritizing sensitivity, whereas a higher threshold may be preferable when minimizing false-positive alerts is more important (9, 10). This behavior is also consistent with the ordinal nature of C-TIRADS itself, which was developed as a structured risk stratification system rather than a simple binary classifier.

A further practical implication of our results concerns the structural completeness of model outputs. ChatGPT-5.4 showed more favorable classification metrics in the primary threshold analysis and produced substantially fewer non-diagnostic outputs than DeepSeek-VL2. From a practical standpoint, this difference is highly relevant. A model that performs reasonably well on interpretable cases but frequently fails to generate a usable category may still be difficult to integrate into clinical workflows. Our results therefore support the view that non-diagnostic rate should be regarded as a meaningful performance metric rather than a peripheral one in AI-assisted diagnostic studies (11).

These findings are strengthened by several methodological features of the study design. First, both models were tested under identical conditions, including the same dual-view image input, the same English prompt, and the same structured output scheme. Second, rather than allowing free-form benign-vs.-malignant judgments, we required structured sonographic feature outputs and estimated C-TIRADS categories, and only then mapped these categories to binary endpoints using predefined thresholds (12). This design improved interpretability and reduced the risk of internal inconsistency between unstructured narrative reasoning and categorical decisions. Third, we retained indeterminate outputs as incorrect classifications in the binary diagnostic analyses, thereby producing conservative full-cohort estimates for these metrics. However, because indeterminate outputs could not be assigned ordinal C-TIRADS risk scores, the ROC analysis excluded these cases and should be interpreted as a valid-output, per-protocol analysis (13).

Several limitations should be considered when interpreting our findings. First, this was a single-center retrospective study with a moderate sample size and included only nodules with pathological confirmation. Because inclusion required pathological confirmation, low-risk nodules that were managed by surveillance without FNA or surgery were not included. This verification process likely contributed to the relatively high malignancy prevalence of 43.2% (89/206), and spectrum bias and verification bias cannot be excluded. Therefore, performance metrics such as accuracy, PPV, and NPV may differ in an unselected thyroid nodule population. Second, 118 initially screened nodules were excluded because of insufficient image quality or missing transverse or longitudinal images. Although these exclusions were necessary for paired-image model evaluation, the present analysis dataset did not contain complete standardized clinical and sonographic variables for all excluded nodules, including age, sex, maximum nodule diameter, and expert C-TIRADS assessment. Therefore, a reliable comparison between included and excluded nodules could not be performed, and potential selection bias remains possible. Third, only static transverse and longitudinal images were evaluated, whereas real-world thyroid ultrasound interpretation often depends on dynamic scanning, Doppler information, elastography, and broader clinical context. Finally, the model-based ROC analysis excluded indeterminate outputs. Because such outputs are unlikely to occur completely at random, the valid-output/per-protocol AUCs are likely to overestimate real-world discrimination, particularly if indeterminate outputs are concentrated among more difficult or lower-quality nodules. In the exploratory comparison of valid-output and indeterminate-output cases, no significant differences were observed for ChatGPT-5.4, whereas DeepSeek-VL2 indeterminate outputs showed a borderline higher proportion of malignant nodules. Therefore, the AUC results should be interpreted together with the non-diagnostic rates and the valid-vs.-indeterminate comparison. In addition, repeat testing was not performed, which may affect the assessment of model stability.

Overall, the present results support a balanced interpretation of general multimodal LLMs in thyroid ultrasound assessment. General multimodal LLMs have already reached a stage at which they can produce structured, partially useful thyroid ultrasound assessments, but their capabilities remain uneven. ChatGPT-5.4 appears closer than DeepSeek-VL2 to the level required for a practical assistive role, yet both models still show clear weaknesses in fine-grained feature recognition and high-consistency risk stratification. At present, they are better viewed as potential adjunctive tools for structured support, education, or workflow standardization than as replacements for expert sonographic interpretation. This interpretation is also consistent with recent thyroid-specific multimodal AI work and with the emerging literature on multimodal GPT systems for thyroid nodule analysis.

## 5 Conclusions

In this study, ChatGPT-5.4 showed numerically better performance than DeepSeek-VL2 in ultrasound-based structured C-TIRADS risk stratification of thyroid nodules, with significantly higher primary-threshold accuracy and specificity, closer agreement with expert C-TIRADS categorization, and a lower non-diagnostic rate. However, the between-model differences in sensitivity and valid-output AUC were not statistically significant, and both models showed limited sensitivity at the primary positive threshold and substantial weaknesses in fine-grained sonographic feature recognition, especially for margin and echogenic foci. Therefore, these models should be regarded as investigational adjunctive tools and should not be used for unsupervised clinical decision-making or standalone triage. Further validation in larger, multicenter datasets and under more clinically realistic imaging conditions is needed before broader clinical deployment can be considered.

## Data Availability

The raw data supporting the conclusions of this article will be made available by the authors, without undue reservation.
