# Supplementary material for: A retrospective comparative study of ChatGPT-5.4 and DeepSeek-VL2 for C-TIRADS-based risk stratification of thyroid nodules on ultrasound images
Source: Front Digit Health. 2026 Jul 23;8:1877420. doi: 10.3389/fdgth.2026.1877420 (PMC13444745; doi:10.3389/fdgth.2026.1877420)
Supplement: Supplementary file 1 [file Datasheet1.docx]

You are evaluating a thyroid nodule on ultrasound.

Two ultrasound images of the same thyroid nodule are provided:

- Image 1: transverse view
- Image 2: longitudinal view

Please analyze BOTH images together as a single lesion. Do not base your judgment on only one image. Your assessment must integrate the findings from the transverse and longitudinal views.

Please provide a structured assessment according to C-TIRADS-related ultrasound features.

Instructions:

1. Evaluate the nodule by integrating both images.
2. Use only the visual information contained in these two ultrasound images.
3. If any feature cannot be confidently determined from the images, state "indeterminate".
4. Do not infer any information not visible in the images.
5. Do not provide a direct benign/malignant diagnosis.
6. Do not provide management recommendations.
7. Output only in the exact format below.
8. If any required feature for C-TIRADS assessment is indeterminate, output "indeterminate" for the Estimated C-TIRADS category.

Output in the following format only:

Composition: [solid / predominantly solid / mixed cystic-solid / cystic / indeterminate]
Echogenicity: [hyperechoic / isoechoic / hypoechoic / markedly hypoechoic / indeterminate]
Orientation: [wider-than-tall / taller-than-wide / indeterminate]
Margin: [smooth / ill-defined / irregular-lobulated / extrathyroidal extension suspected / indeterminate]
Echogenic foci: [none / comet-tail artifact / macrocalcifications / peripheral calcifications / punctate echogenic foci / indeterminate]

Suspicious malignant features identified: [list all identified suspicious malignant features, separated by semicolons; if none, write "none"]

Estimated C-TIRADS category: [2 / 3 / 4A / 4B / 4C / 5 / indeterminate]
